# Enhancing aromatic rice production through agronomic and nutritional management for improved yield and quality

**DOI:** 10.1038/s41598-024-65476-5

**Published:** 2024-07-05

**Authors:** Partha Sarathi Patra, Rajesh Saha, Arju Sahid Ahmed, Bratati Kanjilal, Manoj Kanti Debnath, Bappa Paramanik, Akramul Hoque, Arindam Kundu, Pabitra Adhikary, Amiya Biswas, Prithwiraj Dey, Asim Biswas

**Affiliations:** 1 Regional Research Station, Terai Zone, Uttar Banga Krishi Viswavidyalaya, Pundibari, Cooch Behar, West Bengal 736165 India; 2 Department of Agronomy, Uttar Banga Krishi Viswavidyalaya, Pundibari, Cooch Behar, West Bengal 736165 India; 3 Department of Agricultural Statistics, Uttar Banga Krishi Viswavidyalaya, Pundibari, Cooch Behar, West Bengal 736165 India; 4 Dakshin Dinajpur Krishi Vigyan Kendra, Uttar Banga Krishi Viswavidyalaya, Majhian, Patiram, Dakshin Dinajpur, West Bengal 733133 India; 5 Department of Agronomy, School of Agriculture and Allied Science, The Neotia University, Sarisha, South 24 Parganas, West Bengal 743368 India; 6 North 24 Parganas Krishi Vigyan Kendra, West Bengal University of Animal and Fishery Sciences, Ashokenagar, Haripur, North 24 Parganas, West Bengal 743223 India; 7 Regional Research Station (OAZ), Uttar Banga Krishi Viswavidyalaya, Majhian, Patiram, Dakshin Dinajpur, West Bengal 733133 India; 8 Agricultural and Food Engineeering Department, Indian Institute of Technology Kharagpur, Kharagpur, West Bengal 721302 India; 9 School of Environmental Sciences, University of Guelph, 50 Stone Road East, Guelph, ON N1L 1K2 Canada

**Keywords:** Aromatic rice production, Mechanical rice transplanting, Wet direct seeding, Sustainability, Organic fertilization, Yield and quality enhancement, Soil health, Environmental sciences, Biogeochemistry, Agroecology

## Abstract

To meet the growing international demand for aromatic rice, this study, conducted at Uttar Banga Krishi Viswavidyalaya in Cooch Behar, West Bengal, aimed to enhance the yield and quality of the ‘Tulaipanji’ rice cultivar through advanced establishment methods and the use of organic nutrients over two years. The research tested three planting techniques: mechanical transplanting, wet direct seeding (using a drum seeder), and traditional methods, alongside four nutrient management strategies: vermicompost, farmyard manure, a mix of both, and conventional fertilizers. Findings revealed that mechanical transplanting significantly increased yield by over 31.98% and 71.05% compared to traditional methods and wet direct seeding, respectively. Using vermicompost alone as a nutrient source not only boosted yields by 21.31% over conventional fertilizers but also enhanced the rice's nutritional value and cooking quality. Moreover, soils treated with vermicompost showed higher dehydrogenase activity, indicating better soil health. Economically, mechanical transplanting with vermicompost was the most beneficial, yielding the highest net returns and benefit–cost ratios in both years studied. This approach presents a viable model for improving the sustainability of aromatic rice production globally, emphasizing the economic and environmental advantages of adopting mechanical planting techniques and organic fertilization methods.

## Introduction

Rice (*Oryza sativa* L.), a staple for over half the world’s population^1^, has seen its production systems undergoing tremendous changes to meet the escalating demands for both quantity and quality^2^. Aromatic rice varieties, known for their distinct aroma, superior grain quality, and unique cooking characteristics, hold a prestigious position in the global rice market^3^. In India, aromatic rice such as Basmati and other indigenous scents bear cultural significance and fetch premium prices both domestically and internationally^4,5^. The global preference shift towards higher quality rice, combined with the potential health benefits for individuals with diabetes and obesity, has significantly increased the demand for aromatic rice varieties^6,7^. However, the lower yield of traditional aromatic rice cultivars under conventional cultivation practices poses a major challenge to meeting this growing demand^8^.

India's journey from food scarcity to surplus is a testament to technological adoption and scientific advancement^9^. Yet, the sustainability of aromatic rice production remains an issue due to labor-intensive practices, inefficient nutrient management, and environmental concerns. Manual transplanting, the most prevalent rice establishment method in India, is laborious and time-consuming, accounting for approximately 25% of the crop's total labor requirement^10^. Moreover, it often leads to yield losses due to delays and increases the cost of cultivation. Mechanized transplanting and direct-seeded rice (DSR) are promising alternatives, potentially reducing labor requirements, ensuring timely planting, and lowering the carbon footprint of rice production systems^11^.

Simultaneously, the indiscriminate use of chemical fertilizers in rice cultivation has precipitated not only concerns about soil health and ecosystem sustainability but also the market preference of the rice^12^. Organic agriculture offers a viable pathway to address these challenges by improving rice quality, preserving soil health, and ensuring eco-sustainability^13^. As organic practices enhance the populations of beneficial soil microorganisms, they play a crucial role in nutrient cycling and maintaining soil biological properties^14^. Organically produced rice also exhibited higher price and market demand.

Recognizing the existing methods and practices for aromatic rice cultivation, our study aims to bridge significant knowledge gaps, particularly the trade-offs between yield enhancement and quality retention across various rice establishment techniques and the use of organic nutrient sources. Our research seeks to synthesize these agronomic and nutritional management practices into a streamlined framework, aiming for the sustainable production of aromatic rice. The objectives of the present study are threefold: (1) First, we aim to explore the yield performance and grain quality, including the aroma profile of aromatic rice, under different rice establishment techniques such as manual transplanting, mechanical transplanting, and direct seeding. This will involve a critical evaluation of how these techniques affect both the quantity and quality of rice produced. (2) Second, we intend to assess the effectiveness of organic nutrient sources in improving the nutritional value of aromatic rice, aiming to identify practices that enhance rice quality without compromising yield. (3) Lastly, the study aims to identify the most sustainable combinations of rice establishment and nutrient management practices, analyzing their socio-economic impacts on the livelihoods of farmers in West Bengal and potentially other rice-growing regions in India. By conducting comprehensive field trials and laboratory analyses, this study will fill critical gaps in the literature, offering actionable insights for stakeholders in the journey towards more sustainable aromatic rice production systems that harmonize quality with yield.

## Results

### Performance of agronomic management and grain yield

Table 1 illustrates the agronomic traits affecting yield, such as the number of effective tillers per square meter, panicle length, grains per panicle, filled grains per panicle, and test weight. All yield determinants, except test weight, exhibited significant variations across different establishment methods and nutrient management regimes. The test weight showed no significant changes with either the establishment methods or the nutrient management strategies. Machine transplanting (AM1) led to the highest significant values in average effective tillers per square meter (275.32), grains per panicle (92.01), filled grains per panicle (84.27), and panicle length (23.56 cm) over the two-growing seasons without statistically significant deference between the growing seasons, resulting in a 31.98% and 71.05% higher grain yield compared to the conventional (AM3) and drum seeding methods (AM2), respectively.

**Table 1:** Impact of agronomic and nutrient management options on yield attributes and yield of aromatic rice cultivar Tulaipanji.

Treatments	Effective tillers m^−2^		Panicle length (cm)		No. of grain panicle^−1^		Filled grain panicle^−1^		Test weight (g)		Grain yield (t ha^−1^)	
	2018	2019	2018	2019	2018	2019	2018	2019	2018	2019	2018	2019
Agronomic management options												
AM_1_	268.66	281.98	23.51	23.62	91.62	92.41	83.06	85.48	13.81	13.86	2.59	2.62
AM_2_	208.08	235.95	20.20	20.91	70.01	74.46	60.17	66.37	13.06	13.26	1.44	1.61
AM_3_	226.77	252.05	21.60	21.71	85.41	86.83	73.45	75.16	13.38	13.42	1.88	2.06
SE m (±)	13.20	12.45	0.32	0.27	1.51	1.24	2.04	0.89	0.15	0.18	0.17	0.09
CD (*P* = 0.05)	51.83	48.88	1.27	1.04	5.92	4.88	8.00	3.48	NS	NS	0.68	0.36
Nutrient management options												
NM_1_	244.84	293.31	22.44	24.55	84.07	89.21	73.18	80.8	13.51	13.73	2.13	2.32
NM_2_	220.60	264.49	23.48	19.34	80.48	86.75	75.87	70.76	13.58	13.29	1.82	2.15
NM_3_	275.01	227.06	21.04	22.87	88.16	79.62	71.06	77.44	13.28	13.63	1.90	2.26
NM_4_	197.56	241.77	20.12	21.54	76.68	82.69	68.80	73.68	13.29	13.41	1.65	2.00
SE m (±)	12.64	13.16	0.31	0.29	1.92	1.07	1.56	1.68	0.20	0.12	0.12	0.07
CD (*P* = 0.05)	37.56	39.11	0.93	0.86	5.72	3.17	4.63	5.00	NS	0.36	0.36	0.21
Interaction effects												
SE m ( ±)	21.89	22.80	0.54	0.50	3.33	1.85	2.70	2.92	0.35	0.21	0.21	0.12
CD (*P* = 0.05)	65.05	67.75	1.60	1.49	9.90	5.48	8.03	8.66	1.05	0.62	0.62	0.37

The exclusive use of vermicompost for nitrogen supply (NM1) significantly enhanced the number of average effective tillers per square meter (269.08), grains per panicle (86.64), filled grains per panicle (76.99), and panicle length (23.49 cm) over the two-growing seasons compared to 100% recommended dose of fertilizers (RDF) through inorganic fertilizers (NM4) and the sole application of FYM for nitrogen (NM2). However, a treatment that equally split nitrogen supply between vermicompost and FYM (NM3) yielded statistically similar results. Notably, the application of vermicompost substantially increased average grain yield by 390 and 230 kg ha^−1^ over plots treated solely with chemicals (NM4) and those relying entirely on FYM for nitrogen supply (NM2).

### Milling and cooking quality attributes

The mechanical transplanting method yielded the highest average values for hulling (77.61%), milling (64.74%), head rice recovery (57.61%), starch content (70.25%), and amylose content (23.63%) over the two-growing seasons, with the conventional method (AM3) following closely behind (Table 2). In contrast, plots seeded with the drum method exhibited a higher average amylopectin content of 47.83%. Additionally, providing 100% nitrogen via vermicompost (NM1) resulted in superior average hulling (77.93%), milling (64.57%), head rice recovery (57.40%), starch (72.59%), and amylose (23.54%) values over the two-growing seasons, outperforming the combined application of vermicompost and FYM (NM3) and the sole application of FYM (NM2). The highest average amylopectin content (49.06%) was found in plots that received a 100% chemical treatment (NM4).

**Table 2:** Impact of agronomic and nutrient management options on milling and quality of aromatic rice cultivar Tulaipanji.

Treatments	Hulling (%)		Milling		Head rice recovery (%)		Starch (%)		Amylose (%)		Amylopectin (%)	
	2018	2019	2018	2019	2018	2019	2018	2019	2018	2019	2018	2019
Agronomic management options												
AM_1_	77.52	77.70	64.61	64.87	57.29	57.92	69.80	70.70	23.47	23.80	46.34	46.90
AM_2_	76.48	76.16	62.65	62.51	49.26	50.87	68.63	70.40	21.38	21.98	47.25	48.42
AM_3_	76.71	77.04	63.87	63.98	50.86	50.17	69.02	70.33	22.28	22.90	46.74	47.42
SE m (±)	0.11	0.12	0.15	0.26	0.65	1.20	0.23	0.20	0.64	0.35	0.51	0.44
CD (*P* = 0.05)	0.44	0.45	0.59	1.03	2.57	4.73	0.90	NS	NS	1.36	NS	NS
Nutrient management options												
NM_1_	77.52	78.34	64.26	64.88	57.27	57.52	71.93	73.26	23.36	23.71	45.72	45.97
NM_2_	76.81	76.44	63.5	63.36	50.62	51.13	68.44	69.47	22.01	22.52	46.43	46.95
NM_3_	77.18	76.96	64.1	63.98	52.66	53.94	69.38	71.56	23.00	23.70	46.38	47.86
NM_4_	76.1	76.14	62.97	62.92	49.35	49.35	66.85	67.61	21.14	21.64	48.57	49.54
SE m (±)	0.21	0.22	0.30	0.40	1.59	1.71	0.55	0.37	0.56	0.42	0.72	0.53
CD (*P* = 0.05)	0.64	0.67	0.89	1.20	4.72	5.09	1.63	1.10	1.66	1.25	2.13	1.58
Interaction effects												
SE m (±)	0.37	0.39	0.52	0.70	2.75	2.97	0.95	0.64	0.97	0.73	1.24	0.92
CD (*P* = 0.05)	1.10	1.16	1.54	2.07	8.17	8.82	2.82	1.91	2.87	2.17	3.69	2.73

**Figure 1:**
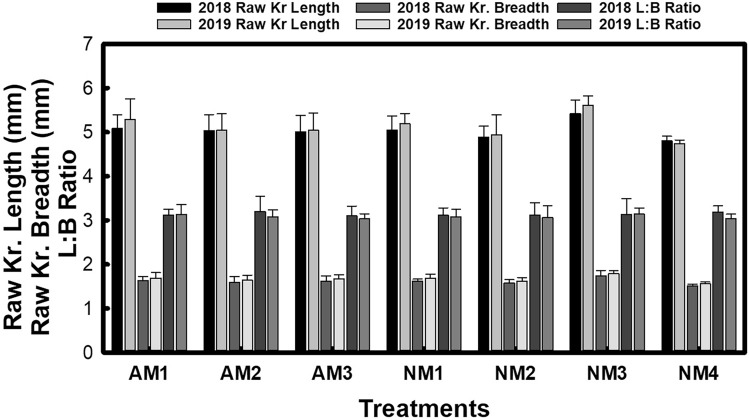
Impact of agronomic and nutrient management options on raw kernel length, breadth and L:B ratio of aromatic rice.

**Figure 2:**
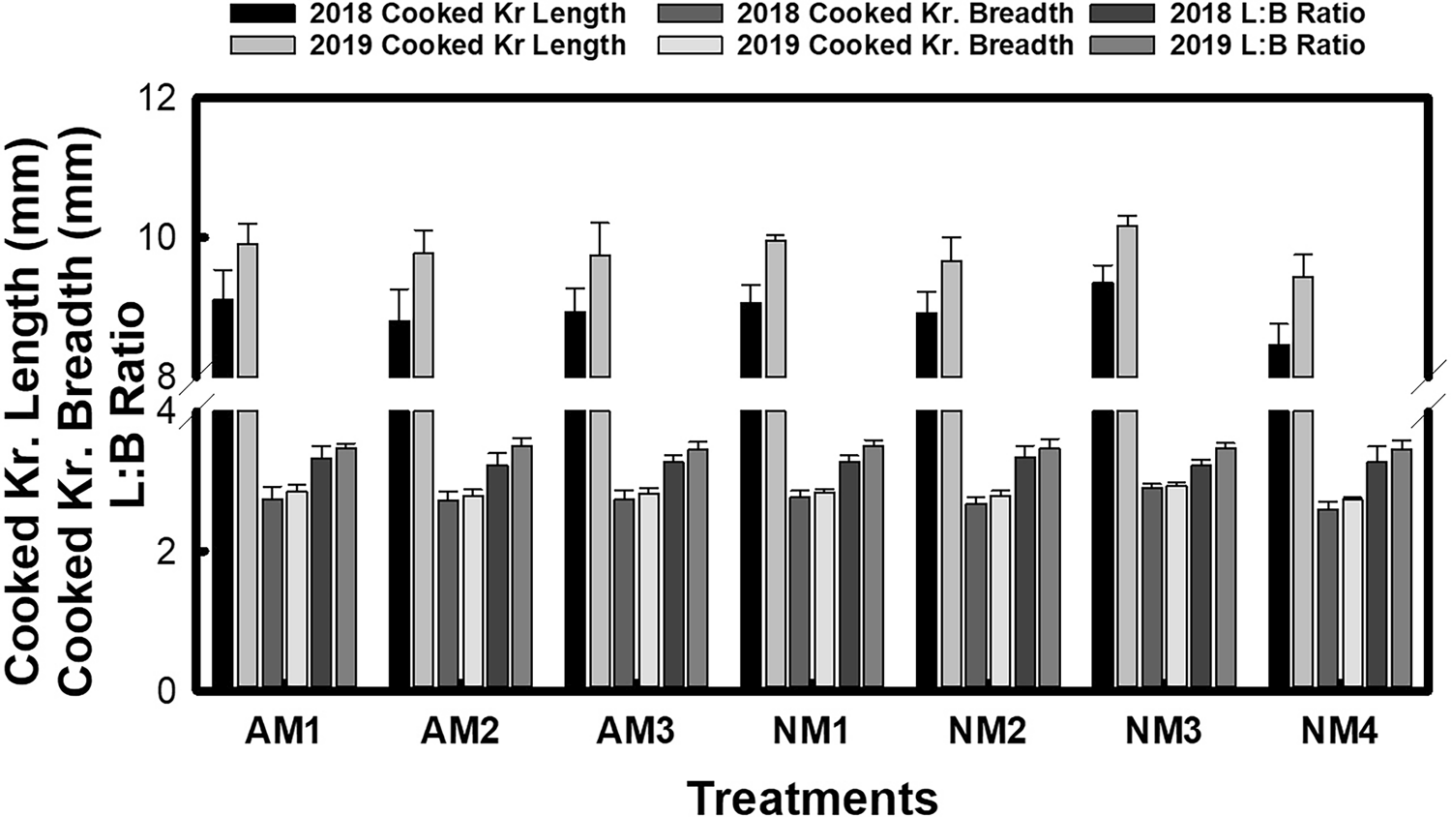
Impact of agronomic and nutrient management options on cooked kernel length, breadth and L:B ratio of aromatic rice.

Kernel dimensions such as average raw length and length-to-breadth (L:B) ratio did not exhibit significant differences among establishment methods, although average kernel breadth showed considerable variation (Fig. 1). Post-cooking kernel length was significantly greater in mechanically transplanted plots, with the highest values for both kernel breadth (2.80 mm, averaged over two growing seasons) and L:B ratio (3.40, averaged over two growing seasons) observed following machine transplantation (Fig. 2). Concerning nutrient management, the highest average kernel dimensions both before and after cooking were achieved when 100% nitrogen was supplied through vermicompost (NM1), followed by the combined vermicompost and FYM treatment (NM3) and the sole FYM treatment (NM2). Inorganic fertilizer alone yielded the lowest average kernel dimensions. The combined influence of establishment methods and nutrient management practices was statistically significant across the two years of study.

### Nutritional composition

The conventional establishment method (AM3) exhibited the highest average moisture content at 10.43%, followed by machine transplantation (AM1) and drum seeding (AM2) (Table 3). Machine transplantation showed the highest average crude protein content (8.83%). Fiber, carbohydrate, ash, and fat concentrations were not significantly affected by the establishment techniques. Among nutrient management treatments, the sole use of vermicompost (NM1) achieved the highest average fiber (0.69%), ash (0.61%), crude protein (8.56%), and carbohydrate (77.70%) contents, translating to the highest average food energy content of 349.75 kcal 100g^−1^. The 100% RDF treatment (NM4) was associated with the highest average grain moisture content (10.18%). The combined effects of the establishment techniques and nutrient management practices were also significant.

**Table 3:** Grain composition of aromatic rice as influenced by agronomic and nutrient management options.

Treatments	Moisture (%)		Fibre (%)		Ash (%)		Crude protein (%)		Carbohydrate (%)		Fat (%)		Food energy (k Cal 100g^−1^)	
	2018	2019	2018	2019	2018	2019	2018	2019	2018	2019	2018	2019	2018	2019
Agronomic management options														
AM_1_	9.77	9.27	0.63	0.65	0.56	0.58	8.72	8.95	75.83	76.56	0.70	0.70	344.47	348.31
AM_2_	9.72	9.01	0.62	0.63	0.55	0.56	7.53	8.08	74.42	75.10	0.71	0.70	334.16	339.04
AM_3_	10.68	10.17	0.63	0.64	0.55	0.56	7.99	8.37	74.59	75.52	0.70	0.69	336.59	341.81
SE m ( ±)	0.14	0.25	0.01	0.01	0.01	0.02	0.17	0.17	0.50	0.83	0.01	0.01	2.27	3.09
CD (*P* = 0.05)	0.55	0.99	NS	NS	NS	NS	0.68	0.68	NS	3.25	NS	NS	8.90	NS
Nutrient management options														
NM_1_	9.94	9.35	0.68	0.70	0.60	0.62	8.13	9.0	76.80	78.60	0.68	0.68	345.20	354.31
NM_2_	10.07	9.57	0.62	0.63	0.53	0.55	7.97	8.43	74.53	75.04	0.71	0.70	335.46	340.33
NM_3_	9.74	9.14	0.63	0.65	0.57	0.58	8.46	7.98	75.46	76.72	0.67	0.66	340.35	348.83
NM_4_	10.48	9.88	0.58	0.58	0.51	0.51	7.75	8.46	73.02	72.53	0.75	0.74	332.62	328.75
SE m (±)	0.18	0.16	0.02	0.02	0.01	0.02	0.11	0.20	0.51	0.94	0.02	0.01	2.19	3.75
CD (*P* = 0.05)	0.53	0.47	NS	0.05	0.04	0.05	0.33	0.61	1.51	2.81	0.05	0.04	6.51	11.15
Interaction effects														
SE m ( ±)	0.31	0.27	0.03	0.03	0.02	0.03	0.19	0.35	0.88	1.64	0.03	0.02	3.79	6.50
CD (*P* = 0.05)	0.92	0.81	NS	0.09	0.07	0.09	NS	1.05	2.61	4.86	0.08	0.07	11.27	19.31

### Soil enzymatic activity and microbial populations

Significant differences in dehydrogenase activity were observed among the establishment techniques (Table 4), with drum-seeded plots exhibiting the highest average enzyme activity (21.86 μg TPF g^−1^ soil h^−1^), followed by mechanically (20.12 μg TPF g^−1^ soil h^−1^) and conventionally transplanted plots (16.94 μg TPF g^−1^ soil h^−1^). For organic treatments, the sole application of vermicompost (NM1) resulted in the highest average dehydrogenase activity (22.44 μg TPF g^−1^ soil h^−1^), which was significantly greater than that obtained with FYM alone (NM2) or a chemical fertilizer regimen (NM4). Similarly, FYM treatments displayed the highest microbial biomass carbon and nitrogen levels.

**Table 4:** Effect of agronomic and nutrient management options on microbial population and dehydrogenase activity.

Treatments	Dehydrogenase activity (μg TPF g^−1^ soil h^−1^)		Microbial population (cfu 10^–5^ g^−1^ of soil)					
			Bacteria		Actinomycetes		Fungi	
	2018	2019	2018	2019	2018	2019	2018	2019
Agronomic management options								
AM_1_	18.80	21.44	23.79	21.83	38.38	43.33	20.50	19.67
AM_2_	20.46	23.27	25.00	28.25	42.67	45.42	23.08	26.25
AM_3_	15.39	18.48	20.83	23.38	44.00	40.79	17.67	23.67
SE m (±)	0.99	0.84	0.57	0.79	0.48	1.17	0.33	0.57
CD (*P* = 0.05)	3.91	3.31	2.24	3.08	1.88	4.58	1.29	2.25
Nutrient management options								
NM_1_	21.73	24.43	25.33	25.33	43.00	44.56	22.44	25.11
NM_2_	17.42	20.01	21.56	24.94	41.44	43.39	18.67	22.89
NM_3_	19.01	21.91	24.06	23.89	42.17	44.00	22.00	22.44
NM_4_	14.70	15.89	21.89	20.78	40.11	40.08	18.56	18.33
SE m (±)	0.71	0.74	0.67	1.10	0.60	1.27	0.53	0.69
CD (*P* = 0.05)	2.10	2.21	2.00	3.26	1.79	3.78	1.58	2.05
Interaction effects								
SE m (±)	1.22	1.29	1.16	1.90	1.04	2.20	0.92	1.19
CD (*P* = 0.05)	3.63	3.83	3.46	5.64	3.10	6.55	2.74	3.54

The combined treatments of establishment methods and nutrient management strategies influenced the soil microbial population significantly. The machine transplanting combined with exclusive vermicompost usage (AM1 + NM1) promoted the highest average bacterial (27.9 × 10^6^ CFU g^−1^ soil), fungal (18.4 × 10^4^ CFU g^−1^ soil), and actinomycetes populations (12.8 × 10^5^ CFU g^−1^ soil), contributing to an enhanced soil health status.

### Nutrient uptake and soil nutrient availability

Regarding nutrient uptake by rice plants, mechanical transplanting (AM1) displayed the most significant extraction of nitrogen, phosphorus, and potassium (NPK), with average values of 68.62, 10.73, and 87.05 kg ha^−1^ respectively. This method was closely followed by the conventional method (AM3), while the drum seeding approach (AM2) showed the least nutrient uptake, as detailed in Table 4. The drum seeded plots exhibited the highest average available soil nitrogen post-cultivation, measuring 133.54 kg ha^−1^, whereas the mechanically transplanted plots recorded the highest average available soil phosphorus (28.09 kg ha^−1^). The available potassium levels were comparable between the mechanical and drum seeding methods. The lowest average available potassium, at 122.40 kg ha^−1^, was observed in plots cultivated using the conventional method.

In terms of nutrient management treatments, the application of 100% nitrogen through vermicompost (NM1) led to the highest plant uptake of NPK (59.13, 9.51, and 75.43 kg ha^−1^, respectively, averaged over two-growing seasons) (Table 5). This was followed by the plots receiving a split application of nitrogen through 50% vermicompost and 50% FYM (NM3), and those receiving 100% nitrogen from FYM alone (NM2). Plots treated with chemical fertilizers (NM4) demonstrated the lowest average nutrient uptake. Interestingly, the highest levels of average available nitrogen post-harvest (136.83 kg ha^−1^) were observed in plots treated with vermicompost (NM1), while the highest average phosphorus (29.52 kg ha^−1^) and average potassium (130.26 kg ha^−1^) levels were found in plots receiving nitrogen solely from FYM (NM2). The lowest nutrient availability post-harvest was noted in soils of the chemically fertilized plots.

**Table 5:** Effect of different establishment techniques and nutrient management options on nutrient uptake and availability.

Treatments	Nutrient uptake (kg ha^−1^)						Nutrient availability (kg ha^−1^)					
	Nitrogen		Phosphorus		Potassium		Nitrogen		Phosphorus		Potassium	
	2018	2019	2018	2019	2018	2019	2018	2019	2018	2019	2018	2019
Agronomic management options												
AM_1_	66.84	70.40	9.98	11.48	86.59	87.50	127.76	127.38	28.73	27.46	121.63	129.67
AM_2_	38.85	42.18	6.11	6.81	57.45	61.26	130.12	136.95	22.40	25.62	120.37	130.18
AM_3_	48.01	51.47	7.29	8.58	63.99	67.51	128.77	128.27	25.15	25.57	122.67	122.13
SE m (±)	3.22	2.11	0.55	0.54	0.79	2.78	1.89	2.59	1.05	0.90	0.81	1.10
CD (*P* = 0.05)	12.64	8.28	2.14	2.13	3.02	10.93	NS	8.08	4.06	NS	NS	3.34
Nutrient management options												
NM_1_	53.57	64.70	8.08	10.95	70.50	80.37	134.81	138.84	26.81	26.91	121.40	125.20
NM_2_	47.95	55.76	7.20	9.28	66.22	74.90	127.81	129.18	29.24	29.81	126.74	133.79
NM_3_	61.36	46.54	9.68	7.32	81.28	63.35	131.91	130.26	23.63	25.26	120.57	127.85
NM_4_	42.05	51.75	6.22	8.29	59.37	69.73	120.50	125.69	22.87	22.02	117.50	122.47
SE m (±)	2.60	3.53	0.43	0.60	2.48	4.58	1.45	2.48	1.31	1.02	1.15	1.25
CD (*P* = 0.05)	7.73	10.51	1.26	1.78	7.37	13.59	4.31	7.36	3.89	3.04	3.42	3.70
Interaction effects												
SE m (±)	4.51	3.53	0.73	0.60	4.29	4.58	2.51	4.29	2.27	1.77	2.00	2.16
CD (*P* = 0.05)	13.39	10.51	2.17	1.78	12.76	13.59	7.46	12.75	6.74	5.26	5.93	6.42

### Economic analysis of the production system

**Figure 3:**
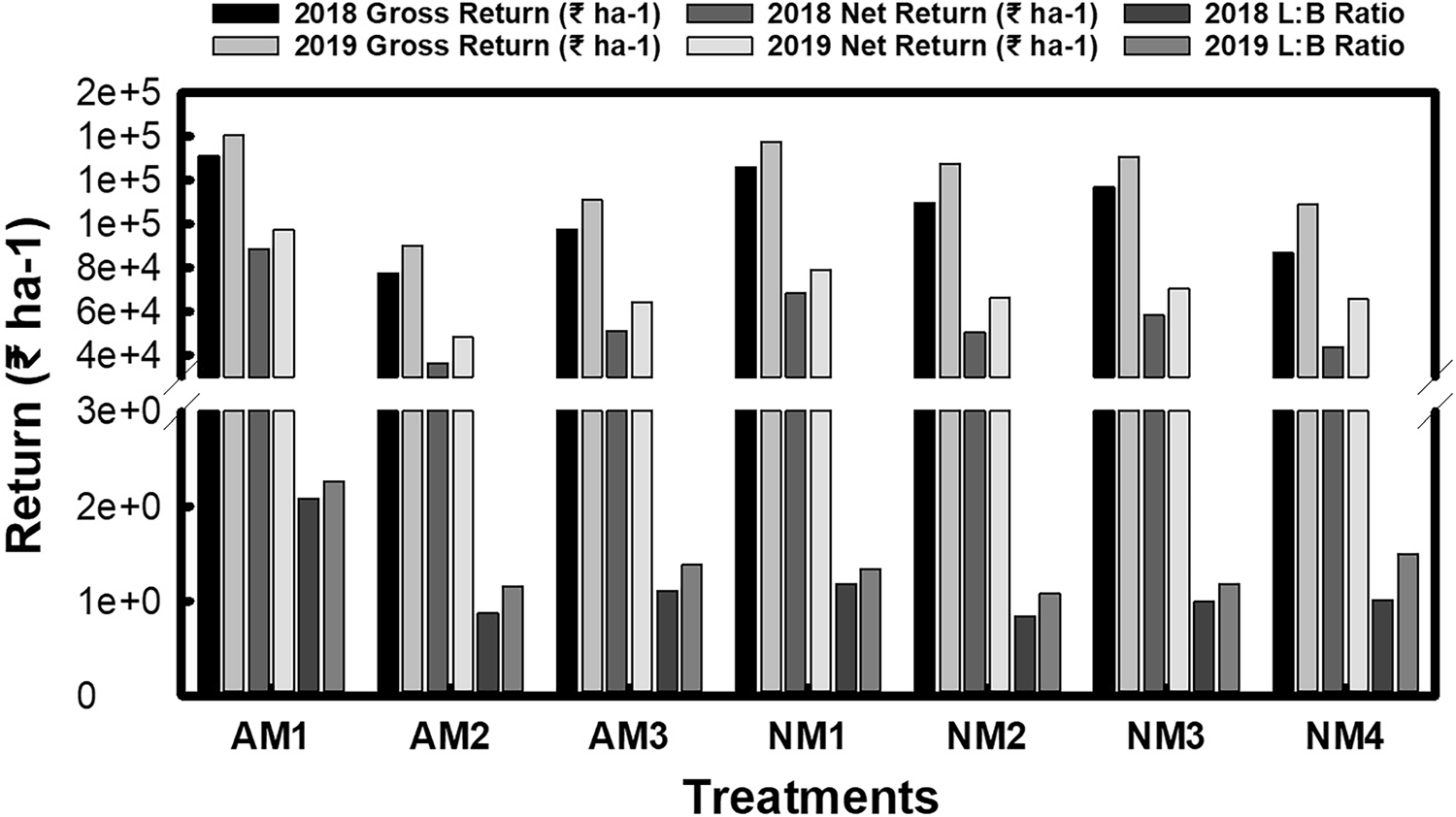
Impact of agronomic and nutrient management options on economics of aromatic rice cultivation.

The economic assessment encompassed both the common costs and specific treatment costs, calculated based on the prevailing prices of inputs and labor. The total cultivation cost was determined by summing the treatment-specific and common costs. The gross returns were then computed by multiplying the economic yield with the prevailing market prices (Fig. 3).

In terms of gross returns, the mechanical method (AM1) emerged as the most profitable, yielding ₹131,140 and ₹140,380 per hectare in 2018 and 2019, respectively (Fig. 3). In contrast, the drum seeding method (AM2) generated the lowest gross returns, at ₹77,240 and ₹89,890 per hectare for the respective years. Among the various nutrient management strategies, the application of 100% nitrogen via vermicompost (NM1) achieved the highest gross returns, recording ₹125,620 and ₹137,400 per hectare in 2018 and 2019, followed by the combined use of vermicompost and FYM (NM3) and the exclusive use of FYM for nitrogen (NM2). The chemically fertilized plots (NM4) demonstrated the lowest gross returns of ₹86,500 and ₹108,880 in 2018 and 2019, respectively.

The net return, which reflects the profit after accounting for costs, varied across the establishment techniques, ranging from ₹35,907 to ₹88,602 in 2018 and ₹48,061 to ₹97,321 in 2019. The mechanical method (AM1) exhibited the highest net return, while the drum seeded method (AM2) showed the lowest. Notably, all organic treatments outperformed the inorganic treatment in terms of net return during both years of the study. Specifically, the application of 100% nitrogen through vermicompost (NM1) was the most profitable among the organic treatments, followed by the split application of nitrogen using FYM and vermicompost (NM3) and the sole use of FYM (NM2). The Benefit-Cost (B:C) ratio was highest for the mechanical method (AM1), registering at 2.08 and 2.26 for 2018 and 2019, respectively. In 2018, the highest B:C ratio of 1.18 was achieved with the vermicompost treatment (NM1), while in 2019, the chemically fertilized treatment (NM4) attained the highest ratio of 1.50.

## Discussion

The global appetite for aromatic rice, with its distinctive flavor profile and nutritional benefits, has ushered in a pressing need for agricultural practices that not only boost yield but also ensure the sustainability of production and the quality of the rice produced^1,3^. Our study, conducted at Uttar Banga Krishi Viswavidyalaya in Cooch Behar, West Bengal, examines the effects of mechanical transplanting versus traditional sowing methods, alongside the utilization of organic versus inorganic nutrient management strategies on the yield and quality of ‘Tulaipanji’ rice. The outcomes of this research, deeply embedded in the practices of sustainable agriculture, have far-reaching implications not just for local farmers but also for the global agricultural community^15,16^.

### Yield attributes and performance

The adoption of mechanical transplanting showcased a notable increase in yield attributes such as the number of effective tillers per square meter and panicle length, directly correlating with the enhanced yield observed. This superior performance is attributed to the precise spacing and depth control afforded by mechanical transplanting, which minimizes transplanting shock and optimizes the young seedlings' growth conditions^17,18^. Additionally, the application of vermicompost, known for its rich nutrient profile and beneficial microbial content, further amplified yield by improving soil health and nutrient availability, thereby supporting robust plant growth^19,20^.

### Grain quality: milling, eating, and cooking attributes

The integration of mechanical transplanting and organic nutrient sources, particularly vermicompost, not only impacts yield but also significantly enhances the milling, eating, and cooking quality of ‘Tulaipanji’ rice. This study found that mechanical transplanting improved hulling and milling percentages, a crucial factor for market acceptance and consumer preference^21,22^. The application of organic nutrients, especially vermicompost, contributed to higher amylose content, which is positively associated with the desirable textural attributes of cooked rice^23,24^. These findings underscore the potential of integrated crop management practices to elevate both the yield and quality of aromatic rice, aligning with consumer demands for premium rice products.

### Nutritional composition and soil health

Exploring the nutritional composition of rice grains, our research indicates that mechanical transplanting and organic nutrient management positively influence the protein and ash content of 'Tulaipanji' rice, suggesting an enhancement in the nutritional value of the grains^25,26^. Furthermore, the increased dehydrogenase activity in soil treated with vermicompost points towards improved soil health, essential for sustainable agricultural practices and long-term soil fertility^14,27^.

### Economic viability

The economic analysis conducted as part of this study highlights the financial benefits of adopting mechanical transplanting and organic nutrient management, particularly vermicompost. This combination not only yielded higher net returns but also exhibited a superior benefit–cost ratio, making it an economically viable option for farmers^28,29^. Such findings are pivotal for encouraging the adoption of these sustainable practices, which promise to enhance profitability while ensuring environmental stewardship.

### Broader implications and future directions

The implications of this research extend beyond the immediate context of ‘Tulaipanji’ rice production, offering valuable insights into sustainable agricultural practices that can be adapted and applied globally. By demonstrating the tangible benefits of mechanical transplanting and organic nutrient management in enhancing yield, grain quality, and economic returns, this study contributes to the broader discourse on sustainable agriculture and food security^30^. It beckons a paradigm shift towards more sustainable crop management practices that hold the promise of addressing the dual challenges of meeting the global food demand and preserving environmental integrity.

In conclusion, the integration of mechanical transplanting and organic nutrient management, particularly through the use of vermicompost, emerges as a compelling strategy for enhancing the productivity, quality, and economic viability of aromatic rice cultivation. This study not only adds to the existing body of knowledge on sustainable rice production but also sets the stage for future research aimed at optimizing these practices for different rice varieties and geographic conditions. As the world grapples with the challenges of food security and environmental sustainability, the findings from this research offer a blueprint for advancing rice cultivation practices that are both productive and sustainable.

## Materials and methods

### Study site

The research was conducted at the Instructional Farm of Uttar Banga Krishi Viswavidyalaya, located in the Terai region of West Bengal, India (26° 19′ 86′′ N, 89° 23′ 53′′ E, 43 m above sea level). This region, with its total geographical area of 1025 sq. km, represents 13.5% of the state’s area. The experimental site was characterized by medium land with efficient drainage, sandy loam soil typical of the Terai region, acidic pH (5.54 to 5.62), high organic carbon content (0.73 to 0.85%), low available nitrogen (124.17 to 129.79 kg ha^−1^) and potassium (121.55 to 122.19 kg ha^−1^) and medium available phosphorus (18.75 to 23.75 kg ha^−1^). Climatic conditions included a rainfall of 1757 mm and 2352 mm for 2018 and 2019 respectively, and an average maximum and minimum temperature range of 33.51–10.75 °C in 2018 and 34.34–10.61 °C in 2019 during the July to December experimental period.

### Experimental design

The study utilized a split-plot design with the main plots comprising three rice establishment techniques or agronomic managements (AMs): AM1 (Mechanical transplanting), AM2 (Wet direct seeding using a drum seeder), and AM3 (Conventional methods as practiced by farmers). A mechanical transplanter was used to transplant rice seedlings grown in a mat type nursery (A1), while the sprouted rice seeds were sown directly on the well-prepared and puddled land using a drum-seeder (AM2). On the other hand, the conventional methods used by farmers (AM3) involve pulling rice seedlings by hand from the nursery bed at the age of 25–30 days and then transplant manually into the puddled and leveled lands with 2–3 seedlings per hill. The variation in the number of seedlings is due to manual operations. The selection of AM1 and AM2 over AM3 was primarily driven by their potential to enhance yield efficiency, reduce labor costs, and improve resource use effectiveness in rice cultivation. These methods not only streamline planting operations and lower environmental impacts compared to traditional practices, but also align with sustainable agricultural goals by optimizing plant growth conditions and reducing methane emissions. Sub-plots were assigned to four nutrient management treatments: NM1 (100% Nitrogen via Vermicompost), NM2 (100% Nitrogen via farmyard manure, FYM), NM3 (50% Nitrogen each from Vermicompost and FYM), and NM4 (Recommended dose of 50:25:25 kg NPK ha^−1^). The use of vermicompost, FYM, and their combination in this study is justified by their well-documented benefits to soil health, including improved nutrient availability and enhanced soil structure, which are vital for sustainable agricultural practices. Vermicompost is particularly valued for its ability to enrich the soil with microorganisms and essential nutrients, leading to increased plant growth and yield; cow dung manure is locally available and traditionally used, making it a cost-effective option for farmers. The combination of these organic amendments is explored due to recent developments that suggest synergistic effects on soil fertility and crop productivity, aligning with the growing interest among local farmers to adopt sustainable and organic farming practices to enhance crop outcomes and soil health sustainably. The aromatic rice cultivar 'Tulaipanji' was sown with 30 × 10 cm spacing during the Kharif season (July–December). Basal fertilization included half of the nitrogen in urea form, with full doses of phosphorous and potassium applied from single super phosphate and muriate of potash, respectively, at final land preparation. The remaining nitrogen was split-applied 35 and 60 days after transplanting/sowing. Organic manures were applied 15 days before transplanting.

### Nutrient composition analysis

The nutrient composition of FYM and vermicompost was determined annually following standard protocols, with results presented in Table 6.

**Table 6:** Nutrient composition organic inputs.

Organic manures	Available N (%)		Available P_2_O_5_ (%)		Available K_2_O (%)	
	2018	2019	2018	2019	2018	2019
Vermicompost	1.74	1.67	0.45	0.51	0.72	0.82
Farmyard manure	0.38	0.48	0.23	0.25	0.25	0.24

### Crop management

Identical crop management practices were maintained across both years. Land preparation involved tractor and power tiller use, followed by the delineation, and leveling of individual plots (24 m^2^ each). Seed treatment was conducted with *Trichoderma viride* at a rate of 5 g kg^−1^ seed. Transplanting and seeding dates varied slightly between the two years as per the specifics listed. Hand weeding occurred at 25, 45, and 65 days after sowing/transplanting. Prophylactic sprays of neem oil @ 5.0 ml liter^−1^ of water at 15 days intervals were carried out to avoid any possible damage by insects and diseases. Fields were irrigated bi-daily to maintain a 5 to 8 cm water layer until 15 days pre-harvest. Harvesting dates were determined by establishment technique and year.

### Data collection and analysis

Yield attributes including effective tillers, panicle numbers, panicle length, grain counts, and test weight were recorded from five randomly selected plants per treatment. Grain and straw yields were quantified from the net plot area and standardized to tonnes per hectare. Quality parameters assessed included carbohydrate, starch, protein, ash, fat, fiber, amylose, amylopectin, moisture, hulling and milling percentages, kernel dimensions, grain elongation ratio, cooking quality, and food energy. Soil and plant samples were analyzed post-harvest for fertility status and nutrient content. Microbial populations and dehydrogenase enzyme activity in soil were also evaluated post-harvest. While the data presented in tables are for individual years, all collected data over the two years were pooled together to carry out statistical analysis to reduce the potential variability in the annual data. While the annual variability in the collected data may exist for a variety of reasons, the focus of the study was to quantify the potential average impact of agronomic and nutritional management on rice yield and quality, thus the statistical analysis focussed on the averaged data over the two growing seasons. Statistical analyses were carried out using SPSS software (version 20). Treatment effects were ascertained via F-test, and significant differences were further analyzed at the 5% probability level. Critical differences, when significant, were computed to aid in the interpretation of treatment effects.

## Conclusion

This study methodically explored the effects of various rice planting techniques and nutrient management strategies on rice production, examining aspects such as growth conditions, grain quality, soil health, and economic viability. The integration of mechanical transplanting with organic manures, particularly vermicompost, was found to enhance several key performance indicators significantly. Our research underscores the potential of mechanical transplanting complemented by organic nutrient sources to substantially benefit rice cultivation worldwide.

Mechanical transplanting was identified as a catalyst for improving grain quality, evidenced by increased hulling, milling, and head rice recovery percentages. The application of organic manures, with a spotlight on vermicompost, was instrumental in providing a balanced nutrient supply. This approach not only enriched the soil with essential nutrients but also fostered improved grain nutrient content and higher crop uptake. Furthermore, the combination of mechanical transplanting with vermicompost was beneficial for soil health, enhancing dehydrogenase enzyme activity and microbial populations, essential markers for soil fertility. Economically, adopting mechanical transplanting and supplementing with vermicompost for nitrogen needs emerged as a highly sustainable and profitable practice. This method yielded superior economic returns and a favorable benefit–cost ratio, advocating its potential as a sustainable agricultural practice.

The findings from this investigation advocate for the adoption of mechanical transplanting and organic nutrient management as a holistic approach to rice production. This strategy not only boosts yield and grain quality but also promotes soil health and economic efficiency. Given rice's vital role in global food security, the widespread adoption of these sustainable practices promises extensive benefits, including enhanced farmer livelihoods and environmental preservation. Encouraging further research and the dissemination of these practices among the global farming community is crucial for realizing the full potential of sustainable agricultural methods in rice production and beyond.

## Data Availability

The data used in this article may be available upon reasonable request via email to the first author (P.S.P.: parthaagro@gmail.com) or the corresponding author (A.B.: biswas@uoguelph.ca).

## Abbreviations

AM: Agronomic management
AM1: Mechanical transplanting
AM2: Wet direct seeding (Drum seeder)
AM3: Conventional methods
NM: Nutrient management
FYM: Farmyard manure
NM1: NM with 100% nitrogen through vermicompost
NM2: Through FYM
NM3: A 50/50 combination of vermicompost and FYM
NM4: The recommended dose of fertilizers
B:C: Benefit-cost ratio
L:B: Length-to-breadth ratio
DSR: Direct-seeded rice
RDF: Recommended doses of fertilizer
CFU: Colony forming unit
C/N: Carbon to nitrogen ratio
